# Worsening of Cardiomyopathy Using Deflazacort in an Animal Model Rescued by Gene Therapy

**DOI:** 10.1371/journal.pone.0024729

**Published:** 2011-09-09

**Authors:** Ida Luisa Rotundo, Stefania Faraso, Elvira De Leonibus, Gerardo Nigro, Carmen Vitiello, Alessio Lancioni, Daniele Di Napoli, Sigismondo Castaldo, Vincenzo Russo, Fabio Russo, Giulio Piluso, Alberto Auricchio, Vincenzo Nigro

**Affiliations:** 1 Telethon Institute of Genetics and Medicine, Napoli, Italy; 2 Institute of Genetics and Biophysics, CNR, Napoli, Italy; 3 A.O. Monaldi, Seconda Università di Napoli, Napoli, Italy; 4 Centro di Biotecnologie, Ospedale A. Cardarelli, Napoli, Italy; 5 Laboratorio di Genetica Medica, Dipartimento di Patologia Generale and CIRM, Seconda Università degli Studi di Napoli, Napoli, Italy; 6 Medical Genetics, Dipartimento di Pediatria, Università degli Studi di Napoli “Federico II”, Napoli, Italy; Virginia Commonwealth University, United States of America

## Abstract

We have previously demonstrated that gene therapy can rescue the phenotype and extend lifespan in the delta-sarcoglycan deficient cardiomyopathic hamster. In patients with similar genetic defects, steroids have been largely used to slow down disease progression. Aim of our study was to evaluate the combined effects of steroid treatment and gene therapy on cardiac function. We injected the human delta-sarcoglycan cDNA by adeno-associated virus (AAV) 2/8 by a single intraperitoneal injection into BIO14.6 Syrian hamsters at ten days of age to rescue the phenotype. We then treated the hamsters with deflazacort. Treatment was administered to half of the hamsters that had received the AAV and the other hamsters without AAV, as well as to normal hamsters. Both horizontal and vertical activities were greatly enhanced by deflazacort in all groups. As in previous experiments, the AAV treatment alone was able to preserve the ejection fraction (70±7% EF). However, the EF value declined (52±14%) with a combination of AAV and deflazacort. This was similar with all the other groups of affected animals. We confirm that gene therapy improves cardiac function in the BIO14.6 hamsters. Our results suggest that deflazacort is ineffective and may also have a negative impact on the cardiomyopathy rescue, possibly by boosting motor activity. This is unexpected and may have significance in terms of the lifestyle recommendations for patients.

## Introduction

Muscular dystrophies are common and debilitating genetic diseases that afflict a significant population of children and adults worldwide [1], [2], [3], [4]. Both skeletal and cardiac muscles are affected. Not only in patients, but also in carriers [5], cardiomyopathy may be the leading cause of death.

In the last two decades multiple disease mechanisms have been discovered. The most relevant involves the impairment of the Dystrophin-glycoprotein complex (DGC) [6].

The DGC plays a central role in maintaining the integrity of the cell membrane by forming a structural link between the extracellular matrix and the cytoskeleton, thus protecting the muscle fibers from contraction-induced damage and necrosis [7].

One member of this complex is delta-sarcoglycan [8] which is a transmembrane glycoprotein and forms a heterotetrameric complex together with alpha-, beta-, and gamma-sarcoglycan [9], [10], [11].

Patients with mutations in the delta-sarcoglycan gene may present with limb girdle muscular dystrophy 2F [12], [13] together with cardiac involvement [14], or isolated cardiomyopathy [15], [16]. The BIO14.6 hamster [17] is an appropriate model for the human disease. It displays an absence of delta-sarcoglycan from the muscle membrane, followed by a deficiency of alpha-, beta- and gamma-sarcoglycan, reproducing the human LGMD2F phenotype. This animal model was generated in 1962 [18], when Homburger fixed by repeated inbreeding a spontaneous trait of the Syrian hamster characterized by muscular dystrophy and cardiomyopathy. The strain carries a homozygous 24-kb deletion of the delta-sarcoglycan gene promoter and the first exon.

At the moment, glucocorticoid treatment [19] is the “gold standard” for Duchenne Muscular Dystrophy (DMD) and sarcoglycan defects and has been shown to increase skeletal muscle strength in DMD patients [20], [21].

The commonly used steroids in published trials are prednisone, prednisolone and deflazacort [22]. The steroid dose used in various trials for prednisolone, or its equivalent glucocorticoid dose, ranges from 0.9 mg to 1.5 mg/kg/day, given daily or on alternate days, or in an intermittent (ten days on, ten or twenty days off) regime.

The precise mechanism by which steroids may increase strength in DMD is not known but their potential beneficial effects include an inhibition of muscle proteolysis [23], a stabilization of muscle fiber membranes [24], an increase in myogenic repair [25], an anti-inflammatory/immunosuppressive effect [26], a reduction of cytosolic calcium concentrations [27], an upregulation of utrophin [28] and a differential regulation of the genes in the muscle fibers [21].

Chronic treatment with deflazacort is also effective in delaying the progression of myocardial fibrosis in *mdx* mice [29]. One study has suggested that anti-inflammatory therapy with oral deflazacort has beneficial effects on the left ventricular function in DMD patients [30]. Another work has shown that long-term deflazacort therapy might be a valuable tool to minimize the progression of cardiomyopathy in DMD patients [29]. However, the effect of corticosteroids on cardiac function in patients with LGMD2C-F remains largely unknown. Bauer *et al.* [31] have shown that in *Sgcd*-null mice prednisolone treatment induces a decompensation of global heart function which is associated with an increase in the myocardial pathology.

Gene vectors derived from adeno-associated viruses (AAV) are among the most promising systems for muscle and heart gene delivery [32], [33]. In the last few years, several studies have shown how a single [34] or a double systemic injection [35] of different AAV pseudotypes can restore the expression of delta-sarcoglycan cDNA in male BIO14.6 hamsters. This treatment was able to rescue the cardiomyopathy and prolong the lifespan in this model. We previously demonstrated that AAVs are well tolerated in hamsters [35] and there is a very weak immune reaction against a specific capsid or delta-sarcoglycan, maybe for the similarity with other sarcoglycans [36].

However, no information is available on the combinatorial therapy including both gene therapy and steroids. In our present research we have tested the effects of long-term deflazacort treatment upon phenotype rescued by gene therapy.

## Results

### Population study

**Figure 1:**
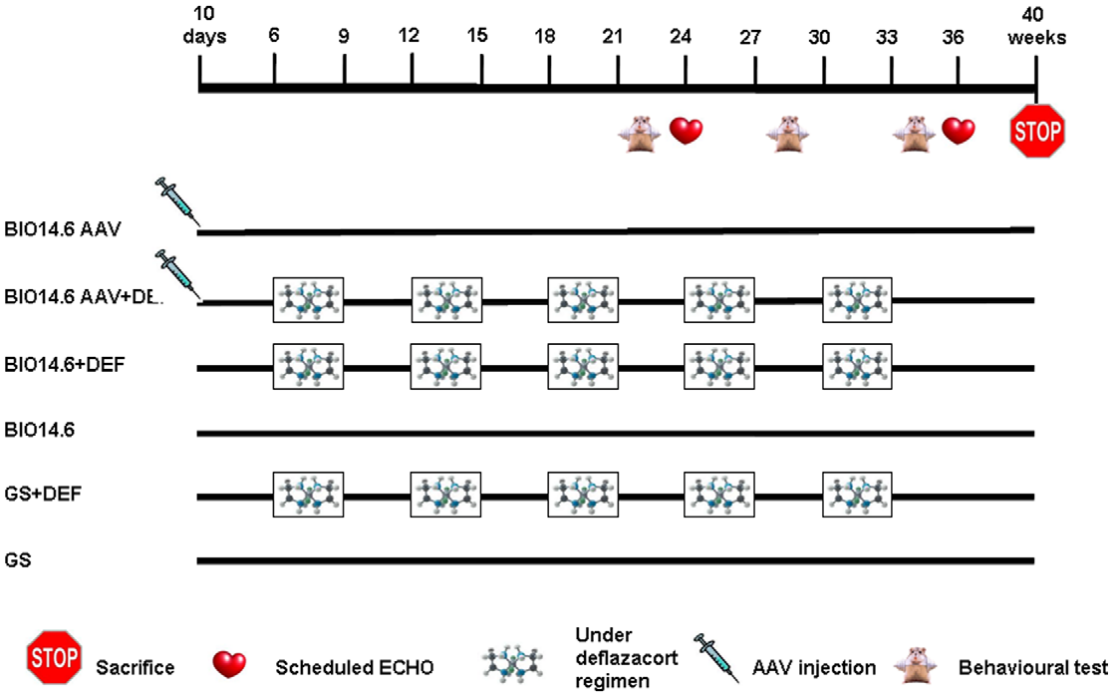
Timeline of the experimental procedures for the different groups of hamsters. BIO14.6 AAV: hamsters injected at 10 days of age with delta-sarcoglycan by AAV2/8; BIO14.6 AAV+DEF: hamsters injected at 10 days of age with delta-sarcoglycan by AAV2/8 and with deflazacort at 6 weeks of age; BIO14.6+DEF: hamsters treated with deflazacort at 6 weeks of age; BIO14.6: hamsters not treated; GS: Golden Syrian hamsters as health control; GS+DEF: Golden Syrian hamsters treated with deflazacort at 6 weeks of age.

To test the combination of gene treatment and corticosteroids, we created six groups of hamsters (Figure 1):

Group BIO14.6AAV (n = 11) were BIO14.6 hamsters intraperitoneally treated at 10 days of age with AAV2/8-CMV-hSCGD without any other treatment.

Group BIO14.6AAV+DEF (n = 11) were BIO 14.6 hamsters intraperitoneally treated at 10 days of age with AAV2/8-CMV-hSCGD and treated starting at 6 weeks of age using 5 cycles of deflazacort for 3 weeks followed by 3 weeks of interval.

Group BIO14.6+DEF (n = 15) BIO14.6 hamsters that were treated starting at 6 weeks of age using 5 cycles of deflazacort for 3 weeks followed by 3 weeks of interval.

Group BIO14.6 (n = 15) BIO14.6 hamsters not treated.

Group GS+DEF (n = 6) controls Golden Syrian hamsters that were treated starting at 6 weeks of age using 5 cycles of deflazacort for 3 weeks followed by 3 weeks of interval.

Group GS (n = 6) controls Golden Syrian hamsters not treated.

### Delta sarcoglycan expression

**Figure 2:**
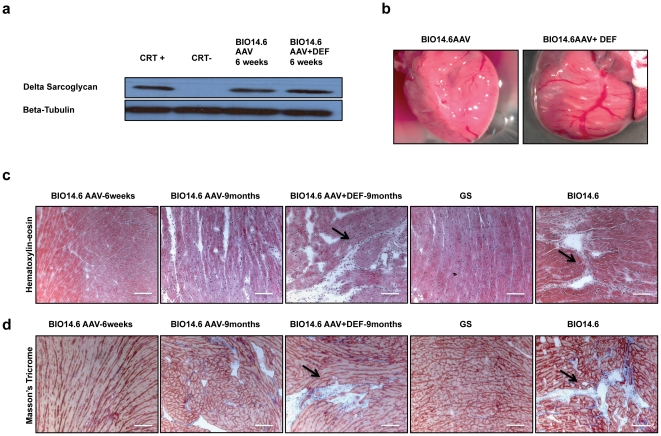
Expression of the injected delta-sarcoglycan and necrotic and fibrotic areas in the heart: (a) Expression of human delta-sarcoglycan assessed by WB analysis in the hearts respectively of a positive control of transduction (CRT+), negative control (CRT-), BIO14.6 AAV and BIO 14.6 AAV+DEF 6 weeks after AAV delivery (∼8 weeks). (b) Explanted hearts from BIO 14.6 AAV and BIO 14.6 AAV+DEF hamsters at 9 months of age. The heart dilation of a BIO14.6AAV+DEF hamster is evident if compared with a BIO14.6 AAV hamster. (c) Hematoxylin-eosin staining on cryosections of the hearts of BIO14.6 AAV 6 weeks after AAV delivery, BIO 14.6 AAV at 9 months, BIO14.6 AAV+DEF at 9 months, the healthy control (GS hamsters) and the untreated BIO14.6 hamsters. (d) On the same groups of hamsters we performed Masson's trichrome staining on a cryosection of the heart. (10 µm).

To test the expression of transgene human delta-sarcoglycan, we analyzed tissue, of some animals, extracts by western blot analysis (Figure 2a), using a monoclonal Ab against human delta-sarcoglycan that cannot detect the hamster protein in wild-type animals because it recognizes a human-specific N-terminal epitope.

Our results show that, similarly to previous results [35], a single injection of AAV8-CMV-hSCGD vector in 10-days-old neonatal BIO14.6 hamsters, achieved a nearly complete gene transfer in the heart. The expression was tested by WB in the BIO14.6AAV group compared with BIO14.6AAV/DEF group, 10 days after starting the first cycle of deflazacort at the dose of 1.5 mg/kg/day (Figure 2a).

### Cardiac muscle pathology

To assess the rescue of the normal cardiac structures we sacrificed animals from BIO14.6AAV and BIO14.6AAV +DEF groups at 9 months of age and removed the hearts. We did not observe any evident dilation in hearts explanted by BIO14.6AAV hamsters, as a further confirmation of the efficacy of the systemic gene therapy[34], [35]. However heart dilation was observed in BIO14.6AAV+DEF animals (Figure 2b). Analogously, sections from all groups of BIO14.6 hamsters showed differences by haematoxylin-eosin (Figure 2c) and Masson's Trichrome staining (Figure 2d) due to the presence of necrotic and fibrotic areas. As expected, GS hamsters were completely normal and DEF treatment did not modify histology (Figure S1).

We identified more fibrotic areas in group BIO14.6 in comparison with BIO14.6 AAV. Again, pathology in BIO14.6AAV+DEF was more similar to BIO14.6 than to BIO14.6AAV.

### Weight and activity

**Figure 3:**
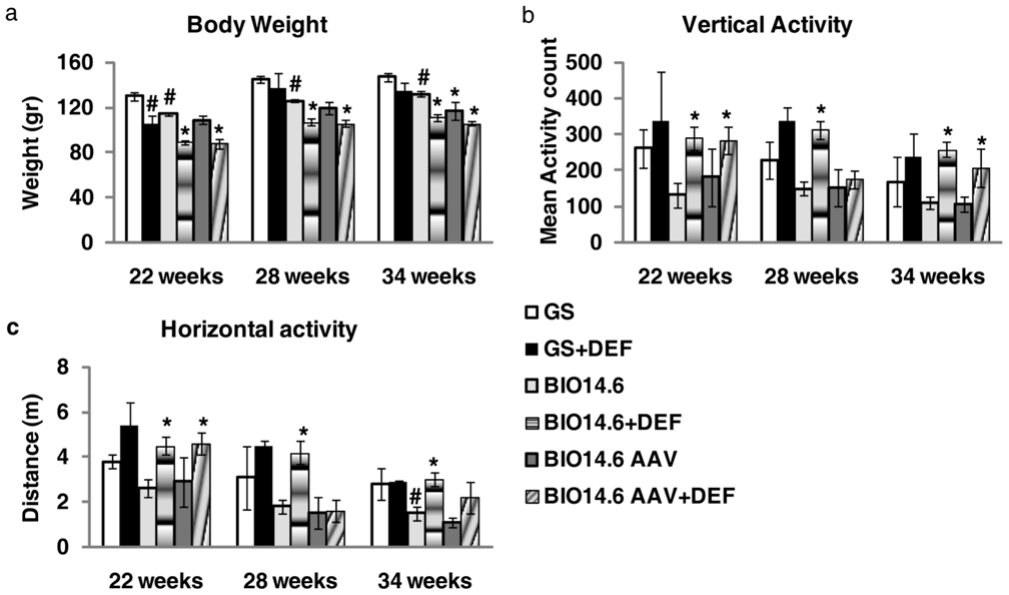
Weight and activity. (a) Body Weight of GS, GS+DEF, BIO14.6, BIO14.6+DEF, BIO14.6AAV and BIO14.6AAV+DEF hamsters in three different test sessions;(b) Vertical activity of GS, GS+DEF, BIO14.6, BIO14.6+DEF, BIO14.6AAV and BIO14.6AAV+DEF hamsters in three different test sessions;(c) Horizontal activity of GS, GS+DEF, BIO14.6, BIO14.6+DEF, BIO14.6AAV hamsters in three different test sessions. # p≥0.05 ; *p<0.05 vs. BIO14.6.

The body weight was reduced in affected hamsters at all time points as compared to normal animals (Figure 3a). Deflazacort treatment further reduced body weight, independently on the age of the animals and on the genotype. No difference in the appetite was observed. In particular weight was reduced of 10–20% between BIO14.6AAV and BIO14.6AAV+DEF, was reduced 20–25% between BIO14.6 and BIO14.6+DEF and also by 10-20% between GS and GS+DEF [Group (F5/33  =  29.502, p<0.0001); Test session (F2/66  =  100.303, p<0.0001); Group x Test session (F10/66  =  2.38, p = 0.017)]. Despite this side effect, prolonged treatment with deflazacort improved all behavioral functions measured in normal and affected animals. As reported in the Figures 3b and 3c, affected hamsters reduced both vertical and horizontal exploratory behavior in a novel environment as compared to normal animals. Animals treated with deflazacort increased vertical activity [Group (F5/31  =  6.47, p = 0.0003); Test session (F2/62  =  4.89, p = 0.01)] and distance traveled in the open field [Group (F5/37 = 3.57, p = 0.0098); Test session (F2/74  =  2.603, p = 0.08)] independently on their age and genotype.

### Cardiac function

**Figure 4:**
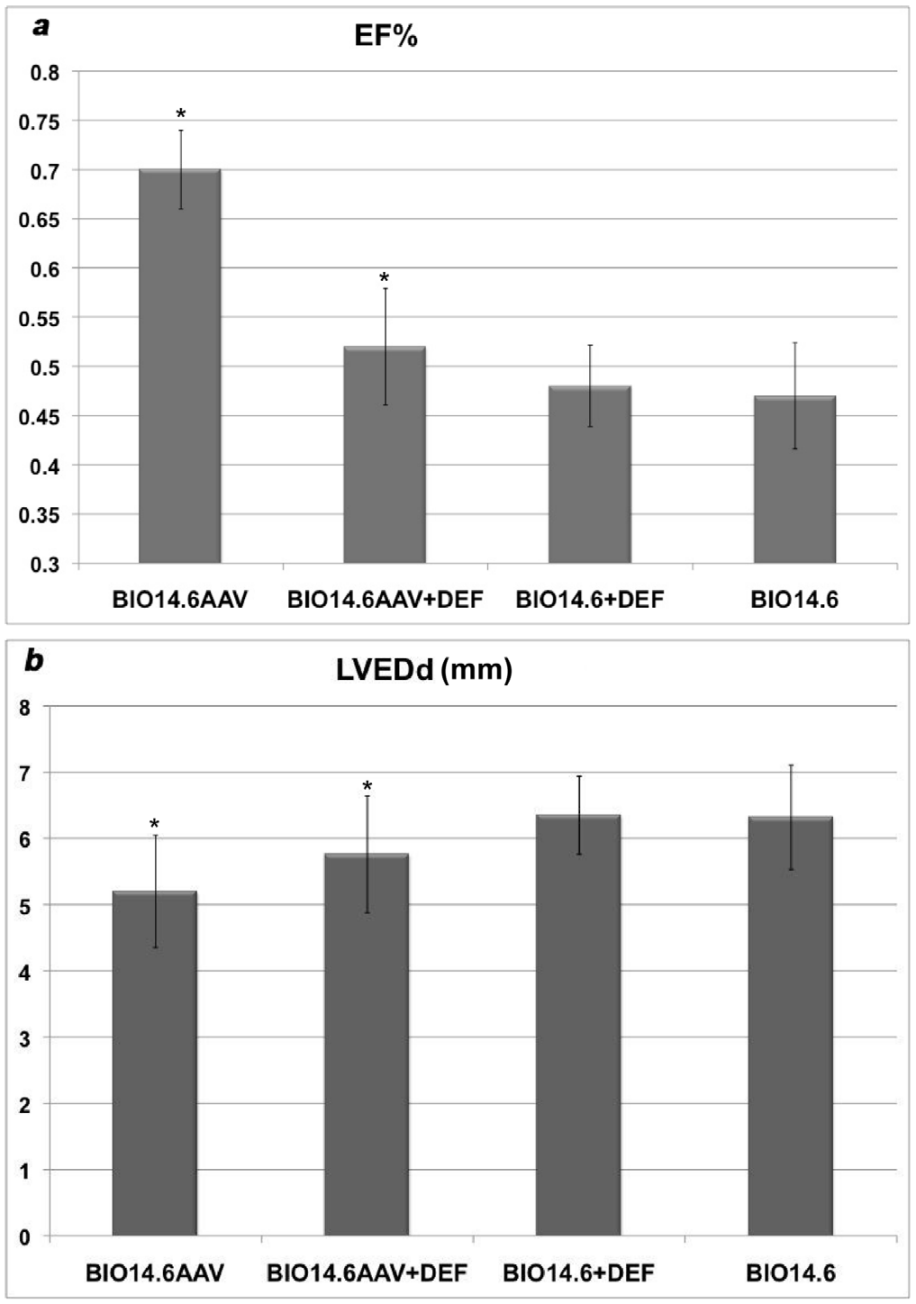
Echocardiographic changes after gene and deflazacort therapy in the different groups of hamsters. (a) ejection fraction (EF) (b) LEVDd respectively in the BIO 14.6 AAV, BIO14.6 AAV+DEF, BIO14.6 +DEF and BIO14.6 hamsters at 9 months. Statistical significance is calculated in comparison with the BIO14.6 group.

None of the animals died during the study. No difference in heart rate was found among the population study. Table 1 shows all measured cardiac parameters values. BIO14.6AAV group showed statistically significant improvement in EF (Fig. 4a) (70±7 *vs* 52±14; 48±6; 47±8%;  = 0,03), FS (35±5 *vs* 23±8; 21±11; 21±15%; p = 0,04), LVEDd (5.20±0.85 *vs* 5.76±0.88; 6.35±0.59; 6.32±0.79 mm; p = 0,03) (Fig. 4b) and LVESd (3.41±0.70 *vs* 4.39±1.13; 5.03±0.80; 5.03±1.01 mm; p = 0,03) with respect to BIO14.6AAV+DEF (n = 5) group, BIO14.6+DEF (n = 11) group and BIO14.6 (n = 12) group respectively. No significant differences were found in all evaluated cardiac parameters between BIO14.6+DEF group and BIO14.6 (p = 0.1). Compared to BIO14.6+DEF group and BIO14.6 group, BIO14.6AAV+DEF group showed increased EF (52±14 *vs* 48±6; 47±8) and reduced LVEDd (5.76±0.88 *vs* 6.35±0.59 *vs* 6.32±0.79). GS hamsters were completely normal and DEF treatment did not modify parameters (Table S1).

**Table 1:** Echocardiographic parameters in the different groups of hamsters at 9 months of age HR =  heart rate; IVST  =  Inter Ventricular Septal Thickness; LVEDd  =  Left Ventricular End Diastolic Diameter; LVESd  =  Left Ventricular End Systolic Diameter; PWT  =  posterior wall thickness; FS  =  Fractional Shortening; EF  =  Ejection fraction.

Parameters	BIO14.6AAV	BIO14.6AAV+DEF	BIO14.6+DEF	BIO14.6
HR (beats/min)	365±20	382±32	389±27	390±30
IVST (mm)	1.16±0.20	1.12±0.13	1.04±0.06	1.11±0.12
LVEDd (mm)	5.20±0.85	5.76±0.88	6.35±0.59	6.32±0.79
LVESd (mm)	3.41± 0.70	4.39±1.13	5.03±0.80	5.03±1.01
PWT (mm)	1.18±0.11	1.33±0.23	1.19±0.11	1.19±0.09
FS (%)	35±5	23±8	21±11	21±15
EF (%)	70±7	52±14	48±6	47±8

## Discussion

The delta-sarcoglycan deficient BIO14.6 hamster is one of the most commonly studied models for inherited dilated cardiomyopathy and muscular dystrophy. In contrast to humans, cardiomyopathy in hamsters occurs with a significantly higher severity than muscular dystrophy and is the cause of premature death at about one year of age. We have previously demonstrated that the systemic transfer of the human delta-sarcoglycan cDNA, by using a combination of AAV vectors [35], brings to the rescue of the BIO14.6 cardiomyopathy and muscular dystrophy. While AAVs are curative for animals, no human patient has been treated thus far by systemic injections at full dosage.

Gene therapy by AAVs has been already used to treat genetic human blindness [37]. At the moment, muscular dystrophies are only treatable in animal models, because there are some inherent difficulties in humans, due to the huge muscle mass, as compared with retina. However, local injections of AAV containing alpha-sarcoglycan have been used successfully [38]. In the next future, improvements in vector preparations and safety will promote clinical trials with systemic injections of AAV. At present, oral steroids are the only effective long-term drug therapy known for human muscular dystrophies. Prolonged ambulation, reduced scoliosis, and improved pulmonary function have been attributed to steroid use [39]. In addition, the use of corticosteroids has also shown the improvement in left ventricular function in Duchenne Muscular Dystrophy (DMD) patients, as previously described [30], [40]. As oral corticosteroids have been used in DMD and in sarcoglycanopathies, we decided to conduct a series of experiments to test the combination of gene therapy and long-term glucocorticoid treatment in the delta-sarcoglycan deficient BIO14.6 hamster. To avoid measuring deleterious side effects caused by steroids, we paid attention to the following points: 1) starting the treatment 6 weeks after birth when the adult sexual development is just complete; 2) using deflazacort instead of prednisone to reduce sodium-retention effects and weight gain [41], [42]; 3) applying a low dose (scaling down to 0.3mg/kg) which should not be immunosuppressive, and 4) adopting an intermittent schedule of three weeks of treatment, followed by three weeks of suspension from the treatment. Most of these points have also been considered in human trials to limit the toxicity and side effects in muscular dystrophy patients.

Following AAV treatment, the expression of the human delta-sarcoglycan was confirmed by western blot analyses. This expression decreased with time, as previously shown, in the absence of a second injection using a different AAV [35]. This was not significant for the present study, since all the animals were sacrificed at 9 months of age, when differences in the lifespan are not yet evident. In fact, at 9 months of age, both the affected and non-affected hamsters were still alive, irrespective of the delta-sarcoglycan mutation. Differences were, however, evident for heart dilation and function, as demonstrated by echocardiography.

In the group of hamsters treated with AAV (BIO14.6AAV group) we found an improvement in the ejection and shortening fraction and a reduction in the left ventricular systolic and diastolic diameters. These parameters are close to those of GS hamsters and a proof of rescue. We found no evident differences in cardiac function and diameters between BIO14.6 group and BIO14.6+DEF group. According to these findings, we show that the deflazacort treatment did not contribute to the cardiomyopathy rescue. This result confirms previous observations with mice, in which prednisolone [31] caused deterioration of the myocardial function and this was probably linked to due to additional mineralocorticoid effects [42] that should be avoided using deflazacort. In contrast the BIO14.6AAV+DEF group, we showed a minimal and not statistically significant change in cardiac function, despite the gene therapy. This result was surprising, since the AAV plus deflazacort treatment reported a phenotype very close to that observed in the affected hamsters that had received no treatment. Deflazacort treatment produced no prompt modification in the expression of delta-sarcoglycan in the heart, as result of a rapid transcriptional inhibition (Figure 2a); in addition, no reduction in the genomic copies of AAV was seen at 9 months (Figure S2), as a result of long-term effects on the AAV turnover. On these bases, we observe that there is no apparent effect of deflazacort on the gene therapy efficacy. However, we cannot exclude the possibility that the steroid treatment can produce a late-onset transcriptional inhibition of the vector promoter in the heart.

The worsened evolution of the cardiomyopathy may have several alternative explanations. It may be due to 1) a possible increase of the animal activity in the cages; 2) an increase in the blood pressure, or 3) water and sodium retention. Our behavioral experiments suggest that the treatment with deflazacort has a strong effect in reducing body weight and intensifying exploratory behavior up to +100%, as well as motility in both BIO14.6 hamsters and control animals. However, further studies are required to evaluate long-term effects by steroids also in other animal models.

We do not know whether this augmented activity can be detrimental in terms of heart failure, but it has been reported that ambulant Becker Muscular Dystrophy patients more often develop a dilated cardiomyopathy and that DMD carriers (with 50% of fibers not expressing dystrophin) have a normal motor activity, although they still develop a cardiomyopathy. An alternative hypothesis is related to increased fluid retention and/or an increase in blood pressure with glucocorticoids. We did not observe statistically significant variations in the pressure, but the average value could be slightly increased for a long time. However, it is important to note that we used a low dosage of deflazacort and the treatment was concluded two months before sacrifice. We cannot exclude that a possible effect of deflazacort on gene therapy may be dependent on increased ER stress and cell stress, but a number of other experiments in further model systems are required to solve this issue.

The improvement obtained by gene therapy can be reversed (totally or partially) by combining gene therapy to glucocorticoid therapy. Is this phenomenon limited to the hamster or can it also be observed in humans? In the former hypothesis, one should consider how much wrong scientific information can derive from studies with animal models. In the latter hypothesis, it may be important to evaluate whether future improvements in the deambulation of muscular dystrophy patients may result in a worsening of the cardiomyopathy.

This is the first study that compares the effect of corticosteroids in animals treated with gene therapy and that demonstrates the harmful effect of the combination of these therapies. Our preferred hypothesis is that the enhancement of activity caused by corticosteroid use and gene therapy together causes an excessive heart stress resulting in an increased risk of cardiac failure.

## Materials and Methods

### AAV vector Construction and production

The human delta-sarcoglycan (SG) gene was cloned into the plasmid pAAV-2.1CMV-EGFP. Recombinant AAV vector containing human delta-sarcoglycan cDNA, driven by the cytomegalovirus (CMV) promoter, was constructed by standard cloning protocols and were packaged into 2/8 serotype. The resulting pAAV2.1-CMV-delta-SG was transfected in sub-confluent 293 cells along with the pAd-Helper and the pack 2/8 packaging plasmid, as described previously [43]. The recombinant AAV2/8 vector was purified by two rounds of CsCl, as described previously. Vector titers, expressed as genome copies per milliliter (GC/ml), were assessed by real-time PCR (ABI 7900 Real Time PCR System), as described previously [44].

### Experimental animals

All the animals were male Syrian hamsters belonging either to strain BIO14.6 (cardiomyopathic) or BIO Golden Syrian Control hamster that is a model for BIO hamster mutants and hybrids. These were purchased from Bio Breeders INC, Fitchburg, Mass (http://www.biobreeders.com) that guarantees homogeneous pure genetic background.

### Ethics Statement

All procedures on wild-type Bio Golden Syrian and dystrophic BIO14.6 hamsters were approved by the “Ministero della Salute” Committee Rome, Italy for “Good Animal experimental Activities”. The investigation conforms to the European Commission Directive 86/609/EEC. Protocol ID 0003092/10/CB.

### Drugs and Devices

Deflazacort (Sanofi-Aventis S.p.A) was administered *per os* with medical food (Mucedola s.r.l). Every three weeks this food was replaced by normal food. We used 1.5 mg/kg in the first cycle, 0.75 mg/kg in the second cycle and 0.3 mg/kg from the third cycle onwards. Both food preparations were humidified to confer a similar appearance and taste. The food was weighed every day.

### Western Blotting

Hamsters were euthanized by inhalation in a CO_2_ chamber by skilled staff. The hearts were rapidly explanted excising the aorta and processed by skilled staff using standard procedure: i.e., they were rinsed in cold phosphate-buffered saline (pH 7.4) containing 0.16 mg/mL heparin to remove red blood cells and clots, frozen in liquid nitrogen, and stored at −70°C. The hearts, muscles or other tissues were homogenized in a lysis assay buffer (Urea 8M, SDS 4%, 125 mM Tris HCl pH 6.8). The samples were separated on sodium dodecyl sulphate - 10% polyacrylamide gel electrophoresis and transferred to nitrocellulose membrane. After blocking in 10% non fat dry milk in Tween-Tris-Buffered Saline (TTBS-1X) buffer (10mM Tris-HCl, 150 mM NaCl, 0.05% Tween 20) for 1 h, the membranes were incubated with primary antibodies in TTBS 1X at room temperature for 1.5 h. The monoclonal antibody recognizing a human epitope of delta-sarcoglycan was used in this experiment with a 1∶25 dilution. Following primary antibody incubation and rinses, the membranes were incubated with the secondary antibody, goat anti-mouse immunoglobulin conjugated with horseradish (Sigma), with 1∶10,000 dilution in 0.5% dry milk and TTBS 1X. After 45 min of antibody incubation and five washes with TTBS 1X buffer, the delta-sarcoglycan protein band was visualized with a chemiluminescence reagent (Supersignal, WestPico, Pierce) and exposed to X-ray film.

### Histology

Tissue samples were collected 6 weeks after AAV injection and at 9 months of age. Samples were processed by cryosections at 7- to 10- µm thickness.

Cryosections of muscular tissues were fixed in 4% PFA, then washed in Phosphate-buffered saline (PBS- 1X) buffer (10 mM Tris-HCl, 200mM NaCl, 0.05% NP 40, 0.05% Tween 20) and stained in haematoxylin for 4 min and in eosin for 6 min. Cryosections were dried in ethanol, fixed in xylene, and mounted with the EUKITT mounting kit (O.Kindler GmbH & CO).

### Masson's trichrome staining

Cryosections of muscular tissues were fixed in Bouin's Solution at 56°C for 15 min, cooled and washed in running tap water to remove the yellow colour from the section. They were stained in Working Weigert's Iron Haematoxylin Solution for 5 min, washed in running tap water for 5 min and stained in Biebrich Scarlet- Acid Fucsin for 5 min, then rinsed in deionised water, placed in Working Phosphotungstic\Phosphomolybdic acid solution for 5 min, stained in Aniline Blue solution for 5 min and in acid acetic 1% for 2 min.

### Behavioral Testing

The behavioral testing room had constant sound and light background. Animals were tested during their light phase, between 9.00 am to 5.00 pm. Before each behavioral task, animals were acclimatized to the testing room for at least 30 min.

General motor activity was measured in an activity cage (Ugo Basile, Italy), with metal floor and Plexiglas transparent walls, covered with white paper. At the beginning of the measurement, each hamster was released from the center of the activity cage. Hamsters were tested for 20 min, during which vertical activity, leaning (standing on the hindlimbs with both forelimbs on the wall) and rearing (standing on the hindlimbs with no support for the forelimbs), was measured by a system of photo beam located on the wall of the cage (Ugo Basile, Italy). Horizontal activity was at the same time automatically scored by a video tracking system (ANY-MAZE, Stoelting, USA) and quantified as distance (m) travelled by the animals in the 20 min interval. Animals were tested during the last week of the deflazacort treatment.

### Statistical analyses

Two-way ANOVA, for repeated measures, with a between-group factor (6 levels: 6 levels: BIO14.6AAV, BIO14.6AAV+DEF, BIO14.6+DEF, BIO14.6, GS+DEF and GS) and test sessions (3 levels: T1, T2, T3) as repeated measure, was used to analyze the body weight, the mean number of vertical activity, and the mean distance travelled. Fisher LSD post hoc test was used to compare the different groups. Statistical significance was set at p<0.05.

### Echocardiographic study

Hamsters were anaesthetized with Isoflurane (Isf) to produce general anesthesia with minimal cardiovascular depression. Isf was administered with the use of a vaporizer and was performed by the same individual over a 1-min period in an isolation chamber with 5.0% Isf in 100% O_2_; the anesthesia was maintained during spontaneous breathing of 1.25% Isf in 100% O_2_ at a flow rate of 1 l/min via a small nose cone. A small plastic bag surrounding the nose cone was attached to wall suction and scavenged excess gas. Left parasternal and left apical echocardiographic images of anaesthetized hamsters lying in the dorsal recumbent position were obtained using the GE Vivid 7 (General Electric, VingMed, Horten, Norway) and an high-frequency probe (30-Mhz) with high frame rate images (80 frames/heartbeat; temporal resolution 2.5 msec). This frame rate is high enough to get reliable velocity and deformation traces. Care was taken to avoid applying excessive pressure, which can induce bradycardia and cardiac arrest. 1-cm region of interest was expanded, allowing high frame rates, and the gain and compression were set for optimal imaging. The chest hair is removed with a topical depilatory agent. All echocardiographic measurements were performed, according to the American Society of Echocardiography recommendations [45], by the same investigator (G.N.) blinded to hamsters clinical status. For intra- and inter-observer variability of analysis, all images were digitally stored and reviewed off-line by and independent observer (V.R.) who had experience in echocardiographic analysis of rodent heart. The three-lead electrocardiogram (ECG) was recorded from the front limbs and the right hind limb. Two-dimensional guided M-mode imaging was used to measure the LV end-systolic (LVESd) and end-diastolic (LVEDd) diameters, interventricular septal thickness (IVST) during diastole and posterior wall thickness (PWT) during diastole, all in the short-axis view at the level of the papillary muscles. The angle of interrogation of the M-mode beam was carefully aligned to be perpendicular to the LV walls at the antero-posterior axis; LVEDd, IVST and PWT were measured by the leading edge method, and the LVESd was measured at the posterior wall's time of maximum anterior motion. Three representative cardiac cycles were analyzed and the mean values for each measurement were recorded. LV systolic function was assessed by ejection fraction (EF; %), calculated from these measurements by an automated computer program which displays the output as EF and by fractional shortening (FS; %) which was calculated from the M-mode echocardiogram using the equation defined as (LVEDd - LVESd)/LVEDd x 100.

## Supporting Information

Figure S1 H-E staining of heart sections of GS and GS +DEF hamsters at 9 months.
(TIF)

Figure S2 Genomic copy number of AAV/mg of tissue extracted from hamster hearts at 9 months of age. The dotted line is the average background value observed with AAV-negative tissue, using real-time PCR.
(TIF)

Table S1 Echocardiographic parameters in GS and GS+DEF hamsters at 9 months of age HR =  heart rate; LVEDd  =  Left Ventricular End Diastolic Diameter; LVESd  =  Left Ventricular End Systolic Diameter; FS  =  Fractional Shortening; EF  =  Ejection fraction.
(DOC)
