# Supplementary figures and images for: Worsening of Cardiomyopathy Using Deflazacort in an Animal Model Rescued by Gene Therapy
Source: PLoS One. 2011 Sep 9;6(9):e24729. doi: 10.1371/journal.pone.0024729 (PMC3170375; doi:10.1371/journal.pone.0024729)

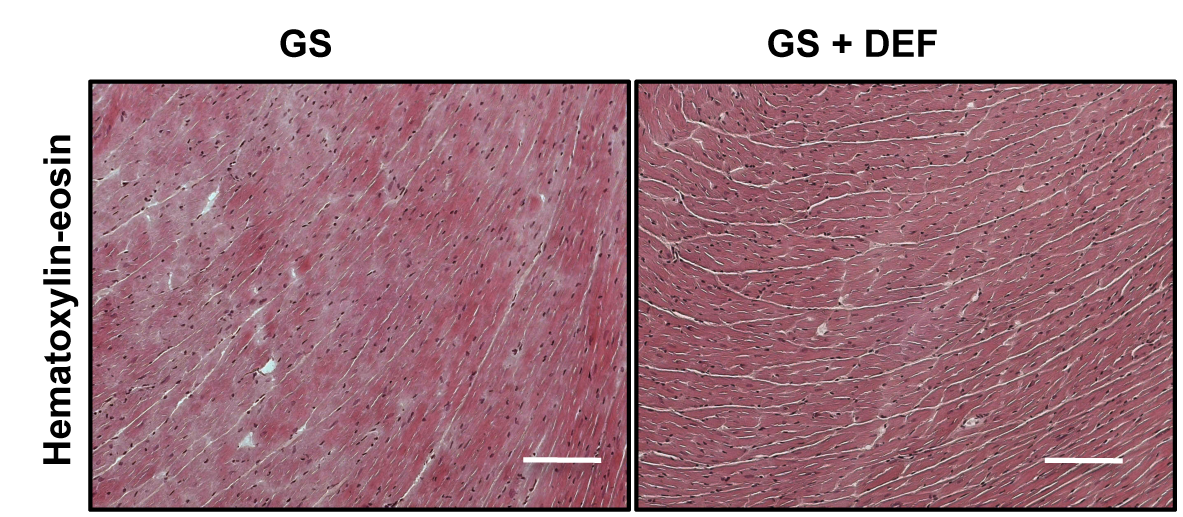

Supplement: Figure S1 — H-E staining of heart sections of GS and GS +DEF hamsters at 9 months. (TIF) [file pone.0024729.s001.tif]

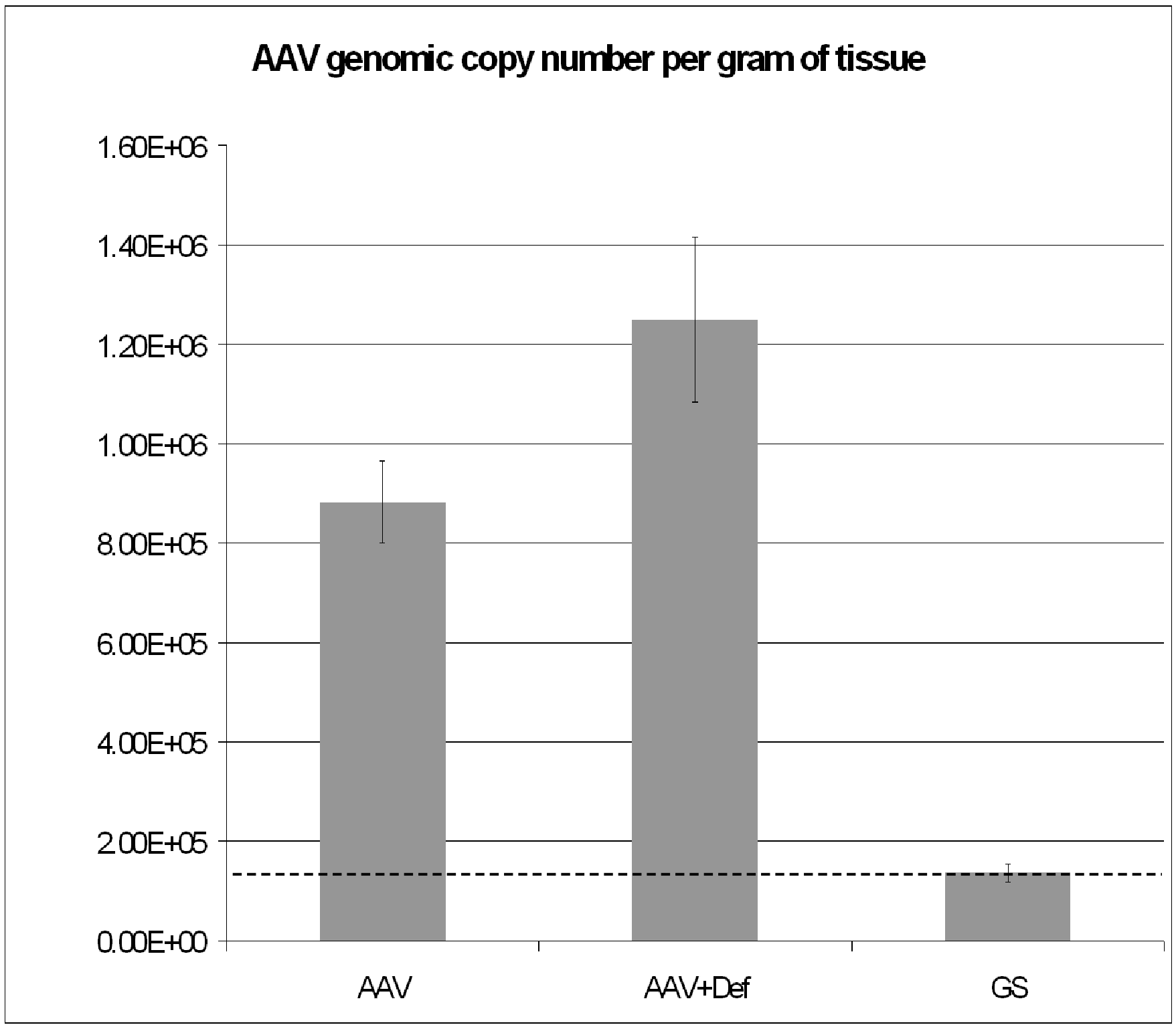

Supplement: Figure S2 — Genomic copy number of AAV/mg of tissue extracted from hamster hearts at 9 months of age. The dotted line is the average background value observed with AAV-negative tissue, using real-time PCR. (TIF) [file pone.0024729.s002.tif]
