# Supplementary material for: Worsening of Cardiomyopathy Using Deflazacort in an Animal Model Rescued by Gene Therapy
Source: PLoS One. 2011 Sep 9;6(9):e24729. doi: 10.1371/journal.pone.0024729 (PMC3170375; doi:10.1371/journal.pone.0024729)
Supplement: Table S1 — Echocardiographic parameters in GS and GS+DEF hamsters at 9 months of age HR = heart rate; LVEDd = Left Ventricular End Diastolic Diameter; LVESd = Left Ventricular End Systolic Diameter; FS = Fractional Shortening; EF = Ejection fraction. (DOC) [file pone.0024729.s003.doc]

| **Parameters** | **GS** | **GS+DEF** |
| --- | --- | --- |
| HR (beats/min) | 370± 20 | 387± 32 |
| LVEDd (mm) | 5.60 ± 0.61 | 5.36 ± 0.92 |
| LVESd (mm) | 3.04 ± 0.33 | 3.02 ± 0.94 |
| FS (%) | 43± 12 | 44 ± 9 |
| EF (%) | 75± 15 | 78 ± 12 |
